# Interplay of EMT and CSC in Cancer and the Potential Therapeutic Strategies

**DOI:** 10.3389/fphar.2020.00904

**Published:** 2020-06-17

**Authors:** Shihori Tanabe, Sabina Quader, Horacio Cabral, Ryuichi Ono

**Affiliations:** ^1^Division of Risk Assessment, Center for Biological Safety and Research (CBSR), National Institute of Health Science (NIHS), Kawasaki, Japan; ^2^Innovation Centre of NanoMedicine (iCONM), Kawasaki Institute of Industrial Promotion, Kawasaki, Japan; ^3^Department of Bioengineering, Graduate School of Engineering, The University of Tokyo, Tokyo, Japan; ^4^Division of Cellular and Molecular Toxicology, Center for Biological Safety and Research (CBSR), National Institute of Health Science (NIHS), Kawasaki, Japan

**Keywords:** cancer stem cell, epithelial-mesenchymal transition, microRNA, nanomedicine, signaling pathway

## Abstract

The mechanism of epithelial-mesenchymal transition (EMT) consists of the cellular phenotypic transition from epithelial to mesenchymal status. The cells exhibiting EMT exist in cancer stem cell (CSC) population, which is involved in drug resistance. CSCs demonstrating EMT feature remain after cancer treatment, which leads to drug resistance, recurrence, metastasis and malignancy of cancer. In this context, the recent advance of nanotechnology in the medical application has ascended the possibility to target CSCs using nanomedicines. In this review article, we focused on the mechanism of CSCs and EMT, especially into the signaling pathways in EMT, regulation of EMT and CSCs by microRNAs and nanomedicine-based approaches to target CSCs.

## Introduction

The cell types transit in human body, which is identified by molecular profiles and contributes into the human disease (Regev et al., 2017). Epithelial-mesenchymal transition (EMT) is defined as cellular phenotypic changes from epithelial to mesenchymal type with high expression of N-cadherin and vimentin, which occurs in various conditions including normal and cancer cells(Tanabe, 2015a; Noh et al., 2017). The EMT plays various roles in the cellular processes such as migration, extracellular matrix (ECM) alteration and apoptosis (Song and Shi, 2018; Peixoto et al., 2019). EMT is also known to drive cell plasticity and contributes in intra-tumor heterogeneity (Krebs et al., 2017; Wahl and Spike, 2017). Cancer poses the entity-specific differences and variety of populations in different malignant stages (Dawood et al., 2014; Fatima et al., 2019). Cancer stem cell (CSC), stem cell population in cancer, is detected with markers such as CD44, while the distinct markers for CSC have not been determined, so far (Yan et al., 2015; Ghuwalewala et al., 2016). Two possibilities for cancer generation, such as the stochastic model and hierarchy model, have been long discussed and are still controversial. CSCs consist of cancer cells with stem-like features, which have capacities of self-renewal, differentiation in cancer cells (Sato et al., 2016). It is also known that some population of CSCs shares the EMT-like cell features (Shibue and Weinberg, 2017). The potential link between EMT and CSCs is a key to cancer drug resistance acquisition, as well as cancer cell plasticity in which the cancer cells transform into the malignant cells and *vice versa* (Loret et al., 2019). To reveal the mechanism of cancer drug resistance, the features of EMT and CSCs should be investigated. The CSCs express transporters on their cell membrane to transport anticancer drugs from inside to outside of the cells (Savage, 2016; Begicevic and Falasca, 2017). The gene and protein expression of the transporters are altered in the CSCs, which may contribute to the acquisition of drug resistance(Lipinska et al., 2017).

## The Role and Regulation of EMT

### The Gene Modules and Network-Based Approaches for EMT-Regulated Genes

EMT controls various cellular processes such as migration, invasion, metastasis, ECM alteration, and apoptosis (Song and Shi, 2018; Peixoto et al., 2019). EMT is implicated in the cancer malignancy, and many genes such as NOTCH family genes are regulated as comparing mesenchymal stem cells (MSCs) and diffuse-type gastric cancer (Tanabe et al., 2015a; Tanabe et al., 2015b). Gene expression of E-cadherin (cadherin1; CDH1) and N-cadherin (cadherin2; CDH2), as well as vimentin are altered in diffuse-type gastric cancer and MSCs (Tanabe et al., 2014). Molecular networks are regulated in EMT, which is a critical process in cancer metastasis and malignancy (Tanabe, 2015b; Tanabe, 2017; Tanabe, 2018a; Tanabe, 2018b; Tanabe et al., 2018). Network-based approach has revealed the several transcription factors predicting diagnosis and drug response in colorectal cancer, which may contribute into the whole understanding of the EMT-regulated mechanisms (Bae et al., 2013; Tanabe, 2018b).

### The Signaling Pathways in EMT

Several signaling pathways such as estrogen receptor signaling, androgen receptor signaling, transforming growth factor beta (TGF-β) signaling and epidermal growth factor (EGF) signaling are involved in EMT in prostate cancer (Montanari et al., 2017). The EMT feature is also involved in resistance in antiandrogen therapy for prostate cancer (Montanari et al., 2017). transforming growth factor beta (TGF-β) signaling, Sonic Hedgehog (SHH) signaling, and WNT signaling pathways are involved in EMT relating development, wound healing and cancer (Zhang et al., 2016). The molecules targeting the signaling related to EMT signaling are anticancer drug candidates, in which the trabedersen (AP12009) inhibiting TGF-β2 expression has been developed for pancreatic cancer treatment, SB431542 inhibiting TGF-β receptor I is used for breast cancer therapy, and LY2109761, another TGF-β receptor inhibitor has been developed for pancreatic cancer treatment (Melisi et al., 2008; Tanaka et al., 2010; Schlingensiepen et al., 2011; Zhang et al., 2016). Fresolimumab, a human anti-TGF-β monoclonal antibody was applied for the treatment in advanced malignant melanoma and renal cell carcinoma patients in Phase I study, which resulted in the acceptable safety and preliminary evidence of antitumor activity (Morris et al., 2014). TGF-β signaling is also targeted in the glioma treatment (Han et al., 2015). It has been revealed that TGF-β signaling and EGF signaling pathways play critical roles in the regulation of the metastasis of aggressive breast cancer (Wendt et al., 2010).

### Regulation of EMT by MicroRNAs

MicroRNAs (miRNAs) are highly conserved, small noncoding, single-stranded RNAs of 20–25 nucleotides that suppress the expression of target genes by translational repression, mRNA degradation, or both (Shyu et al., 2008). To date, 1,917 miRNAs are reported in the human genome (GRCh38) (Kozomara et al., 2019). A single miRNA usually has multiple target genes with partially complementary mRNA sequences, while a single gene can be regulated by several miRNAs (Iorio and Croce, 2012). miRNAs play important roles in the various biological processes, including differentiation, proliferation, apoptosis, and progression of tumors (Zhang and Ma, 2012). In the process of progression of tumors, EMT plays crucial roles in tumor invasion and metastasis. Increasing evidence supports that miRNAs are associated with EMT. A subset of miRNAs (miR-187, miR-34a, miR-506, miR-138, miR-30c, miR-30d, miR-30e-3p, miR-370, and miR-106a) were found to either enhance or suppress the ovarian carcinoma-associated EMT (Koutsaki et al., 2014). Reduced expression levels of the miR-200 family (miR-200a, miR-200b, miR-200c, miR-141, and miR-429) in breast cancer upregulated ZEB1/ZEB2, activating TGF-β/BMP signaling to promote EMT (Saydam et al., 2009). It was reported that the overexpression of miR-200 family could inhibit EMT through the direct suppression of ZEB1/ZEB2 and increases the sensitivity of cancer cells to chemotherapeutic agents (Gregory et al., 2008; Fischer et al., 2015). miR-655 was reported as both an EMT-suppressive miRNA and a predictor for poor prognosis in esophageal squamous cell carcinoma (Harazono et al., 2013). It was also reported that overexpression of miR-509-5p and miR-1243 increased the expression of E-cadherin through the suppression of EMT-related gene expression and that drug sensitivity increased with a combination of each of these miRNAs and gemcitabine (Hiramoto et al., 2017). These reports suggest that miRNAs are one of the promising tools to regulate EMT.

## Linkage Between EMT and CSCs

### Regulation in EMT and CSC Pathways

**Figure 1:**
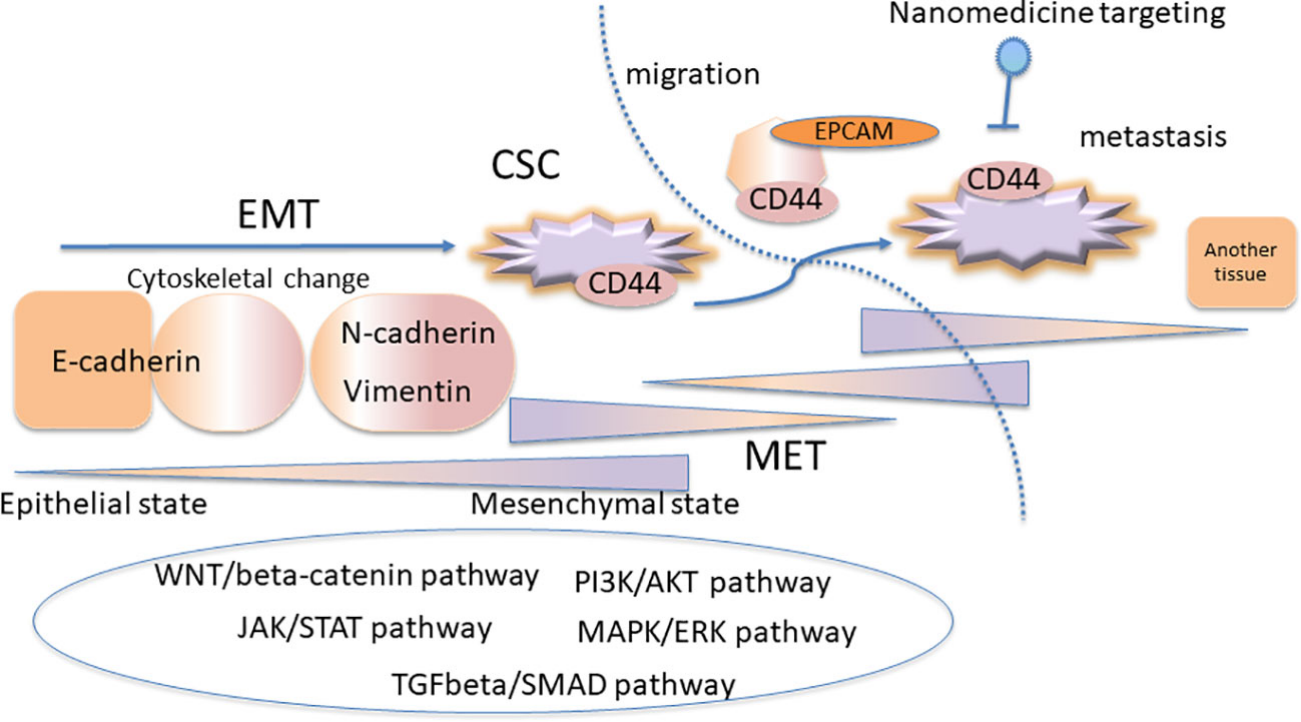
The complexity model scheme for the linkage between epithelial-mesenchymal transition (EMT) and cancer stem cell (CSC) concept. The CSC subpopulations exhibit the EMT phenotypes. The EMT and CSC pathways are regulated at gene level in several signaling pathways, where the plasticity is important for the cancer resistance.

EMT and CSC pathways are regulated at gene level in different pathways such as MAPK/ERK, TGFβ-SMAD, JAK/STAT, PI3K-AKT-NFκB, and WNT/β-catenin pathways (Loret et al., 2019). **Figure 1** shows the complexity model scheme for the linkage between EMT and CSC concept. Diverse genes are involved in cancer phenotypes and heterogeneity, which defines the subtypes of breast cancer (Sørlie et al., 2001). Immune modulatory effect has been reported in EMT and CSCs, which realized the immunotherapy targeting cancer immunity (Deng et al., 2015; da Silveira et al., 2017; Saygin et al., 2019). EMT and CSC properties are involved in the resistance to cytotoxic T lymphocytes (Terry and Chouaib, 2015). Tumor-associated macrophages resides in the microenvironment of the cancer contributes to the EMT characterization (Li et al., 2018). EMT and CSCs are implicated in cellular senescence (Del Barco et al., 2011; Olivos and Mayo, 2016). The cellular senescence can be targeted in terms of the acquisition of stemness of CSCs in cancer therapy (Del Barco et al., 2011; Olivos and Mayo, 2016). The senescence of CSCs is also one of the major reasons of the anticancer treatment which inhibits the cell division (Del Barco et al., 2011; Olivos and Mayo, 2016). The tumor dormancy is one of the major factors of therapy resistance mediated by CSCs (Steinbichler et al., 2018). There is controversially interesting discussion that the dormant tumor cells which acquire EMT phenotypes promote the metastatic proliferation of the cells in CSC-like phenotypes (Weidenfeld et al., 2016). Moreover, in the metastasis process, cytoskeletal changes are required for the cells to migrate from tissues into the blood circulation, which is programmed by TGFβ signaling inducing EMT (Tsubakihara and Moustakas, 2018). The cells exhibiting EMT phenotype demonstrate loosened tight junctions and cell-to-cell adhesion to be ready to migrate (Iqbal et al., 2016; Tsubakihara and Moustakas, 2018).

### The Role of EMT and CSCs in Hypoxia

Hypoxia is involved in the development and aggressiveness of solid tumors. The hypoxia-inducible factor (HIF), which is a main transcriptional regulator of cellular responses to hypoxia, regulate hypoxia responsive genes and contributes to increased cell proliferation, survival, angiogenesis, invasion and metastasis, as well as resistance to therapies. The EMT and CSCs are associated with the hypoxia in tumors, which plays an important role in the regulation and maintenance of the CSC phenotype. It has been demonstrated that the *in vitro* exposure of several human carcinoma cell lines induces EMT, likely due to mechanisms associated with HIF-1 activation (Imai et al., 2003; Lester et al., 2007; Yang et al., 2008). Moreover, it has been reported that HIF-1 promotes EMT of carcinoma cells in clear cell renal cell carcinoma, suppressing E-cadherin indirectly by inducing the expression of ZEB1 and ZEB2 and E2A immunoglobulin enhancer-binding factors E12/E47 (TCF3). Such inhibition leads to the mesenchymal characteristics to the carcinoma (Esteban et al., 2006; Krishnamachary et al., 2006). These findings indicate the complex contribution of various factors contributing to EMT in carcinoma cells, and represent a formidable challenge for formulating therapeutic approaches to control the EMT in tumors.

### Plasticity in EMT and CSCs

CSCs are also phenotypically heterogeneous both inter- and intratumorally (Magee et al., 2012; Meacham and Morrison, 2013), which poses a significant challenge for developing targeted therapies. The heterogeneity of CSCs could be given by genetic mutations and epigenetic changes, or by microenvironmental differences, such as cell-cell interaction, cytokines, and hypoxia (Magee et al., 2012; Meacham and Morrison, 2013). Increasing evidence is also indicating that CSCs exist in anatomically and physiologically specialized environments within tumors, constituting niches that favor their survival (Plaks et al., 2015; Brozovic, 2017). The reliance of the CSCs on niche signals is a general phenomenon and has been demonstrated in several tumors (Borovski et al., 2011; Plaks et al., 2015). CSCs can in turn modulate their niche, and utilize cell-signaling pathways for maintaining homeostatic processes, such as inflammation, EMT, hypoxia and angiogenesis (Borovski et al., 2011; Plaks et al., 2015). Consequently, the architecture and position of this niche are dynamic, and change with tumor development and progression, as well as with the applied treatments. Additionally, the microenvironment of the niche can revert nontumorigenic cancer cells into CSCs by EMT-associated processes, increasing tumor invasion and metastasis (Visvader and Lindeman, 2008; Borovski et al., 2011; Iliopoulos et al., 2011; Plaks et al., 2015). Such dynamic interchange between cancer cells and CSC population suggests that therapies that are only active against CSCs may eventually result in the recurrence/resistance, if the residual differentiated cancer cells can repopulate the niche of CSCs. The cells with the mesenchymal phenotype release from the tissues to enter the circulation as circulating tumor cells (CTCs), where the mesenchymal-epithelial transition (MET) may occur to express the epithelial marker EPCAM in the blood circulation condition. However, it is still controversial that CTCs having high expression of EPCAM correlate with low survival, as shown in castration-resistant prostate cancer patients (de Wit et al., 2018). CTCs themselves may have a variety of subpopulations (Onidani et al., 2019). Therefore, targeted therapeutics aimed for tumor suppression should be able to reach the entire tumor, including CSC niches, at any step of tumor development and eliminate both cancer cells and CSCs with sufficient selectivity, for achieving safe and robust long-term responses.

### Targeting EMT and CSCs by miRNAs

EMT and CSC formation have strong correlations in tumor cell invasion and metastasis. Moreover, EMT and CSCs are related to the acquisition of chemoresistance of tumor cells. Recently, miRNAs were reported to suppress chemoresistance in CSCs. For example, overexpression of miR-608, which targets ribonucleotide reductase large subunit M1 (RRM1) and cytidine deaminase (CDA), decreased the viability of the gemicitabine-resistant MIA-PaCa-RG4 and AsPC-RG2 pancreatic cancer cells (Rajabpour et al., 2017). Overexpression of miR-204 significantly inhibited the metastasis and invasion of gastric cancer cells through the suppression of EMT by SNAI1 (Liu et al., 2016). miR-361-3p regulates ERK1/2-induced EMT *via* direct negative regulator of DUSP2 mRNA degradation in pancreatic ductal adenocarcinoma (Hu et al., 2018). Overexpression of miR-195-5p in DLD1 and HCT116 colon cancer cells repressed cell growth, colony formation, invasion, and migration, while the inhibition of miR-195-5p function contributed to aberrant cell proliferation, migration, invasion, and EMT (Sun et al., 2017). The emerging reports related to miRNA regulation may support the targeting EMT and CSCs by miRNAs (Tanabe and Ono, 2018).

## Therapeutic Strategies for CSC Targeting

### Potential Therapeutic Strategies to Target CSCs

The resistance of CSCs to conventional chemo- and radio-therapies, along with intratumoral heterogeneity and associated plasticity are the significant factors contributing to cancer treatment failure and recurrence. Accordingly, therapies that target CSCs and interrelated cellular hierarchy, represent an attractive direction toward developing robust cancer cures. Among different CSC targeting strategies, meddling with CSC surface markers, controlling drug efflux channels and transporters and targeting signaling pathways, such as Wnt, Hedgehog, and Notch pathways, produced encouraging pre-clinical results and are now in different phases of clinical trials (Koury et al., 2017; Annett and Robson, 2018; Desai et al., 2019; Saygin et al., 2019). Drug-induced differentiation to convert CSCs to non-CSCs (Moreb et al., 2017; van Gils et al., 2017), or inducing MET (Pattabiraman et al., 2016; Pattabiraman and Weinberg, 2016) has also been experimentally confirmed as a practical therapeutic approach to sensitize this therapy-resistant subpopulation to chemotherapies. Several differentiation-inducing agents have shown their vast potential as in single-agent anticancer therapies in pre-clinical studies, however, equivalent responses have not always been observed in patients. These epigenetic modulators generally exert pleiotropic effects and have relatively low cytotoxicity on malignant cells; accordingly, their true therapeutic potential most likely lies in optimal combination with other anticancer drugs (Bayat Mokhtari et al., 2017). In this respect, concomitant cytotoxic therapy with differentiation therapy is expected to prove as a promising therapeutic approach to eradicate this tumor-driving cell population and ultimately bring complete remission. In addition to the differentiation-inducing CSC targeting strategies, immunotherapy-based anti-CSC approaches are also receiving much research attention. Considering that co-inhibitory molecules and immune checkpoint ligands, such as programmed death-ligand 1 (PD-L1) and programmed death-ligand 2 (PD-L2), are highly expressed on CSCs of various cancers, many research groups have also evaluated immunotherapeutic approaches to target CSCs. Among different immunotherapy-based strategies that have been assessed to target CSCs, adaptive T-cells, dendritic cell (DC)-based vaccines, oncolytic viruses, and immune checkpoint inhibitors are typical examples and have recently been reviewed elaborately by Badrinath and Yoo (Badrinath and Yoo, 2019; Saygin et al., 2019). Added to the strategies mentioned above; another rational approach of targeting CSCs is signified by the use of nanotechnology as an efficient tool for the detection and elimination of CSCs. Nanotechnology-based approach will be discussed elaborately in the following chapter.

### Nanomedicines Targeting CSCs

Nanotechnologies for disease management has already demonstrated significant potential in clinical oncology with several anticancer drug-loaded nanomedicines being already in clinical use (Tran et al., 2017; Aftab et al., 2018). With a unique pharmacokinetics (PK)/pharmacodynamics (PD) profile, nanomedicine delivers many pharmacological advantages over conventional chemotherapy, such as improved bioavailability, reduced toxicity, and increased target-tissue selectivity (Rodallec et al., 2018). Nanomedicine has the potential for developing unprecedented therapies by controlling drug functions in a spatiotemporal manner (Cabral et al., 2018), which allows for effective targeting to subpopulations in tumor tissues, as well as intracellular therapeutic targets. With CSC subpopulation in tumors accounting for resistance to therapies and tumor recurrence, institution of novel therapeutic strategies capable of eradicating the CSC fraction is central for achieving the robust responses and long-term patient survival. In this respect, nanomedicine has the potential to target CSCs for realizing effective cancer treatment outcome. For example, the polymeric micelle-based nanomedicine incorporating cisplatin (CDDP/m), which is currently in Phase III of clinical trials for treating pancreatic cancer (Cabral and Kataoka, 2014), was able to eradicate both undifferentiated cell and differentiated cancer cell populations within tumors by controlled intratumoral and intracellular navigation (Wang et al., 2016). This effect was demonstrated in head and neck cancers, where a majority of late-stage cancer cases suffer tumor relapse following cisplatin treatment. Additionally, several nanomedicine-type approaches for both CSC-related therapy and diagnostics have been developed recently (Reda et al., 2019). Combination of thermo and chemotherapy utilizing multifunctional magnetic nanoparticles have been used to target CSCs for the effective cancer treatment (Liu et al., 2020). The nanomedicine containing miR-125b-5p to target EMT and CSCs effectively demonstrated the tumor inhibition *in vivo* (Guo et al., 2019). Another nanomedicine strategy includes co-loaded liposomes of cabazitaxel and CSC inhibitor silibinin to target CD44 receptors on CSCs (Mahira et al., 2019). Strategies capable of enhancing the levels of nanomedicines in tumors and cancer cells could also promote the activity in CSC-rich tumors. Besides peptides or small molecules, the surface of nanomedicines can be modified with other ligands, such as antibodies, antibody fragments or aptamers, providing a versatile platform for targeting tumors and CSCs.

## Conclusion

The CSCs exhibit a variety of features, some of which demonstrate EMT characteristics. CSCs contribute into the anticancer drug resistance leading to the recurrent of cancer, which may be one of the targets for the therapy. Signaling pathways in EMT and CSCs are possible targets for cancer treatment. Considering that these pathways in EMT and CSCs are also found in normal cells, specific targeting of the cancer is critical. There have been emerging evidences that miRNAs regulate various cellular phenotypic changes including EMT and CSCs, which may be one of the targets for cancer therapy. Varieties of novel therapeutics, including nanomedicine and immunotherapy, also have great possibilities for CSC targeting. Further advancement of CSC therapies in combination with several targeting strategies can be envisioned in the near future.

## Author Contributions

ST contributed conception and design of the organization of the review. ST, SQ, HC, and RO wrote the first draft of the sections of the manuscript. All authors contributed to the article and approved the submitted version.

## Funding

This work was supported by Japan Agency for Medical Research and Development (AMED) under Grant Number JP20ak0101093 (ST and RO) and JP20mk0101163 (RO), and Strategic International Collaborative Research Program [JP20jm0210059 (SQ and ST)], Ministry of Health, Labour, and Welfare (MHLW), (H30-KAGAKU-IPPAN-002) (ST and RO), and JSPS KAKENHI (18K19315) (RO).

## Conflict of Interest

The authors declare that the research was conducted in the absence of any commercial or financial relationships that could be construed as a potential conflict of interest.

## References

[B1] Aftab S. Shah A. Nadhman A. Kurbanoglu S. Aysil Ozkan S. Dionysiou D. D. (2018). Nanomedicine: An effective tool in cancer therapy. Int. J. Pharm. 540 (1-2), 132–149. 10.1016/j.ijpharm.2018.02.007 29427746

[B2] Annett S. Robson T. (2018). Targeting cancer stem cells in the clinic: Current status and perspectives. Pharmacol. Ther. 187, 13–30. 10.1016/j.pharmthera.2018.02.001 29421575

[B3] Badrinath N. Yoo S. Y. (2019). Recent Advances in Cancer Stem Cell-Targeted Immunotherapy. Cancers (Basel) 11 (3), 310. 10.3390/cancers11030310 PMC6468501 30841635

[B4] Bae T. Rho K. Choi J. W. Horimoto K. Kim W. Kim S. (2013). Identification of upstream regulators for prognostic expression signature genes in colorectal cancer. BMC Syst. Biol. 7 (1), 86. 10.1186/1752-0509-7-86 24006872 PMC3847874

[B5] Bayat Mokhtari R. Homayouni T. S. Baluch N. Morgatskaya E. Kumar S. Das B. (2017). Combination therapy in combating cancer. Oncotarget 8 (23), 38022–38043. 10.18632/oncotarget.16723 28410237 PMC5514969

[B6] Begicevic R. R. Falasca M. (2017). ABC Transporters in Cancer Stem Cells: Beyond Chemoresistance. Int. J. Mol. Sci. 18 (11), 2362. 10.3390/ijms18112362 PMC5713331 29117122

[B7] Borovski T. De Sousa E. M. F. Vermeulen L. Medema J. P. (2011). Cancer stem cell niche: the place to be. Cancer Res. 71 (3), 634–639. 10.1158/0008-5472.CAN-10-3220 21266356

[B8] Brozovic A. (2017). The relationship between platinum drug resistance and epithelial-mesenchymal transition. Arch. Toxicol. 91 (2), 605–619. 10.1007/s00204-016-1912-7 28032148

[B9] Cabral H. Kataoka K. (2014). Progress of drug-loaded polymeric micelles into clinical studies. J. Controlled Release 190, 465–476. 10.1016/j.jconrel.2014.06.042 24993430

[B10] Cabral H. Miyata K. Osada K. Kataoka K. (2018). Block Copolymer Micelles in Nanomedicine Applications. Chem. Rev. 118 (14), 6844–6892. 10.1021/acs.chemrev.8b00199 29957926

[B11] da Silveira W. A. Palma P. V. B. Sicchieri R. D. Villacis R. A. R. Mandarano L. R. M. Oliveira T. M. G. (2017). Transcription Factor Networks derived from Breast Cancer Stem Cells control the immune response in the Basal subtype. Sci. Rep. 7 (1), 2851. 10.1038/s41598-017-02761-6 28588211 PMC5460106

[B12] Dawood S. Austin L. Cristofanilli M. (2014). Cancer stem cells: implications for cancer therapy. Oncology. (Williston Park) 28 (12), 1101–1107, 1110. 25510809

[B13] de Wit S. Manicone M. Rossi E. Lampignano R. Yang L. Zill B. (2018). EpCAM(high) and EpCAM(low) circulating tumor cells in metastatic prostate and breast cancer patients. Oncotarget 9 (86), 35705–35716. 10.18632/oncotarget.26298 30479699 PMC6235023

[B14] Del Barco S. Vazquez-Martin A. Cufi S. Oliveras-Ferraros C. Bosch-Barrera J. Joven J. (2011). Metformin: multi-faceted protection against cancer. Oncotarget 2 (12), 896–917. 10.18632/oncotarget.387 22203527 PMC3282095

[B15] Deng Z. Wu Y. Ma W. Zhang S. Zhang Y.-Q. (2015). Adoptive T-cell therapy of prostate cancer targeting the cancer stem cell antigen EpCAM. BMC Immunol. 16 (1), 1. 10.1186/s12865-014-0064-x 25636521 PMC4318439

[B16] Desai A. Yan Y. Gerson S. L. (2019). Concise Reviews: Cancer Stem Cell Targeted Therapies: Toward Clinical Success. Stem Cells Transl. Med. 8 (1), 75–81. 10.1002/sctm.18-0123 30328686 PMC6312440

[B17] Esteban M. A. Tran M. G. B. Harten S. K. Hill P. Castellanos M. C. Chandra A. (2006). Regulation of E-cadherin Expression by *VHL* and Hypoxia-Inducible Factor. Cancer Res. 66 (7), 3567–3575. 10.1158/0008-5472.CAN-05-2670 16585181

[B18] Fatima N. Srivastava A. N. Nigam J. Raza S. T. Rizvi S. Siddiqui Z. (2019). Low Expression of MicroRNA335-5p Is Associated with Malignant Behavior of Gallbladder Cancer: A Clinicopathological Study. Asian Pac J. Cancer Prev. 20 (6), 1895–1900. 10.31557/APJCP.2019.20.6.1895 31244315 PMC7021618

[B19] Fischer K. R. Durrans A. Lee S. Sheng J. Li F. Wong S. T. (2015). Epithelial-to-mesenchymal transition is not required for lung metastasis but contributes to chemoresistance. Nature 527 (7579), 472–476. 10.1038/nature15748 26560033 PMC4662610

[B20] Ghuwalewala S. Ghatak D. Das P. Dey S. Sarkar S. Alam N. (2016). CD44highCD24low molecular signature determines the Cancer Stem Cell and EMT phenotype in Oral Squamous Cell Carcinoma. Stem Cell Res. 16 (2), 405–417. 10.1016/j.scr.2016.02.028 26926234

[B21] Gregory P. A. Bert A. G. Paterson E. L. Barry S. C. Tsykin A. Farshid G. (2008). The miR-200 family and miR-205 regulate epithelial to mesenchymal transition by targeting ZEB1 and SIP1. Nat. Cell Biol. 10 (5), 593–601. 10.1038/ncb1722 18376396

[B22] Guo R. Wu Z. Wang J. Li Q. Shen S. Wang W. (2019). Development of a Non-Coding-RNA-based EMT/CSC Inhibitory Nanomedicine for In Vivo Treatment and Monitoring of HCC. Advanced Sci. (Weinheim Baden-Wurttemberg Germany) 6 (9), 1801885–1801885. 10.1002/advs.201801885 PMC6498119 31065520

[B23] Han J. Alvarez-Breckenridge C. A. Wang Q. E. Yu J. (2015). TGF-beta signaling and its targeting for glioma treatment. Am. J. Cancer Res. 5 (3), 945–955. 26045979 PMC4449428

[B24] Harazono Y. Muramatsu T. Endo H. Uzawa N. Kawano T. Harada K. (2013). miR-655 Is an EMT-suppressive microRNA targeting ZEB1 and TGFBR2. PloS One 8 (5), e62757. 10.1371/journal.pone.0062757 23690952 PMC3653886

[B25] Hiramoto H. Muramatsu T. Ichikawa D. Tanimoto K. Yasukawa S. Otsuji E. (2017). miR-509-5p and miR-1243 increase the sensitivity to gemcitabine by inhibiting epithelial-mesenchymal transition in pancreatic cancer. Sci. Rep. 7 (1), 4002. 10.1038/s41598-017-04191-w 28638102 PMC5479822

[B26] Hu J. Li L. Chen H. Zhang G. Liu H. Kong R. (2018). MiR-361-3p regulates ERK1/2-induced EMT via DUSP2 mRNA degradation in pancreatic ductal adenocarcinoma. Cell Death Dis. 9 (8), 807. 10.1038/s41419-018-0839-8 30042387 PMC6057920

[B27] Iliopoulos D. Hirsch H. A. Wang G. Struhl K. (2011). Inducible formation of breast cancer stem cells and their dynamic equilibrium with non-stem cancer cells via IL6 secretion. Proc. Natl. Acad. Sci. U. S. A. 108 (4), 1397–1402. 10.1073/pnas.1018898108 21220315 PMC3029760

[B28] Imai T. Horiuchi A. Wang C. Oka K. Ohira S. Nikaido T. (2003). Hypoxia attenuates the expression of E-cadherin via up-regulation of SNAIL in ovarian carcinoma cells. Am. J. Pathol. 163 (4), 1437–1447. 10.1016/S0002-9440(10)63501-8 14507651 PMC1868286

[B29] Iorio M. V. Croce C. M. (2012). MicroRNA dysregulation in cancer: diagnostics, monitoring and therapeutics. A comprehensive review. EMBO Mol. Med. 4 (3), 143–159. 10.1002/emmm.201100209 22351564 PMC3376845

[B30] Iqbal W. Alkarim S. AlHejin A. Mukhtar H. Saini K. S. (2016). Targeting signal transduction pathways of cancer stem cells for therapeutic opportunities of metastasis. Oncotarget 7 (46), 76337–76353. 10.18632/oncotarget.10942 27486983 PMC5342819

[B31] Koury J. Zhong L. Hao J. (2017). Targeting Signaling Pathways in Cancer Stem Cells for Cancer Treatment. Stem Cells Int. 2017, 2925869. 10.1155/2017/2925869 28356914 PMC5357538

[B32] Koutsaki M. Spandidos D. A. Zaravinos A. (2014). Epithelial-mesenchymal transition-associated miRNAs in ovarian carcinoma, with highlight on the miR-200 family: prognostic value and prospective role in ovarian cancer therapeutics. Cancer Lett. 351 (2), 173–181. 10.1016/j.canlet.2014.05.022 24952258

[B33] Kozomara A. Birgaoanu M. Griffiths-Jones S. (2019). miRBase: from microRNA sequences to function. Nucleic Acids Res. 47 (D1), D155–D162. 10.1093/nar/gky1141 30423142 PMC6323917

[B34] Krebs A. M. Mitschke J. Lasierra Losada M. Schmalhofer O. Boerries M. Busch H. (2017). The EMT-activator Zeb1 is a key factor for cell plasticity and promotes metastasis in pancreatic cancer. Nat. Cell Biol. 19, 518. 10.1038/ncb3513 28414315

[B35] Krishnamachary B. Zagzag D. Nagasawa H. Rainey K. Okuyama H. Baek J. H. (2006). Hypoxia-Inducible Factor-1-Dependent Repression of *E-cadherint* in von Hippel-Lindau Tumor Suppressor–Null Renal Cell Carcinoma Mediated by TCF3, ZFHX1A, and ZFHX1B. Cancer Res. 66 (5), 2725–2731. 10.1158/0008-5472.CAN-05-3719 16510593

[B36] Lester R. D. Jo M. Montel V. Takimoto S. Gonias S. L. (2007). uPAR induces epithelial-mesenchymal transition in hypoxic breast cancer cells. J. Cell Biol. 178 (3), 425–436. 10.1083/jcb.200701092 17664334 PMC2064849

[B37] Li S. Xu F. Zhang J. Wang L. Zheng Y. Wu X. (2018). Tumor-associated macrophages remodeling EMT and predicting survival in colorectal carcinoma. OncoImmunology 7 (2), e1380765. 10.1080/2162402X.2017.1380765 29416940 PMC5798198

[B38] Lipinska N. Romaniuk A. Paszel-Jaworska A. Toton E. Kopczynski P. Rubis B. (2017). Telomerase and drug resistance in cancer. Cell Mol. Life Sci. 74 (22), 4121–4132. 10.1007/s00018-017-2573-2 28623509 PMC5641272

[B39] Liu Z. Long J. Du R. Ge C. Guo K. Xu Y. (2016). miR-204 regulates the EMT by targeting snai1 to suppress the invasion and migration of gastric cancer. Tumour. Biol. 37 (6), 8327–8335. 10.1007/s13277-015-4627-0 26729198

[B40] Liu D. Hong Y. Li Y. Hu C. Yip T.-C. Yu W.-K. (2020). Targeted destruction of cancer stem cells using multifunctional magnetic nanoparticles that enable combined hyperthermia and chemotherapy. Theranostics 10 (3), 1181–1196. 10.7150/thno.38989 31938059 PMC6956796

[B41] Loret N. Denys H. Tummers P. Berx G. (2019). The Role of Epithelial-to-Mesenchymal Plasticity in Ovarian Cancer Progression and Therapy Resistance. Cancers (Basel) 11 (6), 838. 10.3390/cancers11060838 PMC6628067 31213009

[B42] Magee J. A. Piskounova E. Morrison S. J. (2012). Cancer stem cells: impact, heterogeneity, and uncertainty. Cancer Cell 21 (3), 283–296. 10.1016/j.ccr.2012.03.003 22439924 PMC4504432

[B43] Mahira S. Kommineni N. Husain G. M. Khan W. (2019). Cabazitaxel and silibinin co-encapsulated cationic liposomes for CD44 targeted delivery: A new insight into nanomedicine based combinational chemotherapy for prostate cancer. BioMed. Pharmacother. 110, 803–817. 10.1016/j.biopha.2018.11.145 30554119

[B44] Meacham C. E. Morrison S. J. (2013). Tumour heterogeneity and cancer cell plasticity. Nature 501 (7467), 328–337. 10.1038/nature12624 24048065 PMC4521623

[B45] Melisi D. Ishiyama S. Sclabas G. M. Fleming J. B. Xia Q. Tortora G. (2008). LY2109761, a novel transforming growth factor beta receptor type I and type II dual inhibitor, as a therapeutic approach to suppressing pancreatic cancer metastasis. Mol. Cancer Ther. 7 (4), 829–840. 10.1158/1535-7163.MCT-07-0337 18413796 PMC3088432

[B46] Montanari M. Rossetti S. Cavaliere C. D'Aniello C. Malzone M. G. Vanacore D. (2017). Epithelial-mesenchymal transition in prostate cancer: an overview. Oncotarget 8 (21), 35376–35389. 10.18632/oncotarget.15686 28430640 PMC5471062

[B47] Moreb J. S. Ucar-Bilyeu D. A. Khan A. (2017). Use of retinoic acid/aldehyde dehydrogenase pathway as potential targeted therapy against cancer stem cells. Cancer Chemother. Pharmacol. 79 (2), 295–301. 10.1007/s00280-016-3213-5 27942929

[B48] Morris J. C. Tan A. R. Olencki T. E. Shapiro G. I. Dezube B. J. Reiss M. (2014). Phase I study of GC1008 (fresolimumab): a human anti-transforming growth factor-beta (TGFbeta) monoclonal antibody in patients with advanced malignant melanoma or renal cell carcinoma. PloS One 9 (3), e90353. 10.1371/journal.pone.0090353 24618589 PMC3949712

[B49] Noh M. G. Oh S. J. Ahn E. J. Kim Y. J. Jung T. Y. Jung S. (2017). Prognostic significance of E-cadherin and N-cadherin expression in Gliomas. BMC Cancer 17 (1), 583. 10.1186/s12885-017-3591-z 28851312 PMC5575836

[B50] Olivos D. J. Mayo L. D. (2016). Emerging Non-Canonical Functions and Regulation by p53: p53 and Stemness. Int. J. Mol. Sci. 17 (12), 1982. 10.3390/ijms17121982 PMC5187782 27898034

[B51] Onidani K. Shoji H. Kakizaki T. Yoshimoto S. Okaya S. Miura N. (2019). Monitoring of cancer patients via next-generation sequencing of patient-derived circulating tumor cells and tumor DNA. Cancer Sci. 110 (8), 2590–2599. 10.1111/cas.14092 31169336 PMC6676129

[B52] Pattabiraman D. R. Weinberg R. A. (2016). Targeting the Epithelial-to-Mesenchymal Transition: The Case for Differentiation-Based Therapy. Cold Spring Harb. Symp. Quant. Biol. 81, 11–19. 10.1101/sqb.2016.81.030957 28057845 PMC5722631

[B53] Pattabiraman D. R. Bierie B. Kober K. I. Thiru P. Krall J. A. Zill C. (2016). Activation of PKA leads to mesenchymal-to-epithelial transition and loss of tumor-initiating ability. Science 351 (6277), aad3680. 10.1126/science.aad3680 26941323 PMC5131720

[B54] Peixoto P. Etcheverry A. Aubry M. Missey A. Lachat C. Perrard J. (2019). EMT is associated with an epigenetic signature of ECM remodeling genes. Cell Death Dis. 10 (3), 205. 10.1038/s41419-019-1397-4 30814494 PMC6393505

[B55] Plaks V. Kong N. Werb Z. (2015). The cancer stem cell niche: how essential is the niche in regulating stemness of tumor cells? Cell Stem Cell 16 (3), 225–238. 10.1016/j.stem.2015.02.015 25748930 PMC4355577

[B56] Rajabpour A. Afgar A. Mahmoodzadeh H. Radfar J. E. Rajaei F. Teimoori-Toolabi L. (2017). MiR-608 regulating the expression of ribonucleotide reductase M1 and cytidine deaminase is repressed through induced gemcitabine chemoresistance in pancreatic cancer cells. Cancer Chemother. Pharmacol. 80 (4), 765–775. 10.1007/s00280-017-3418-2 28887583

[B57] Reda A. Hosseiny S. El-Sherbiny I. M. (2019). Next-generation nanotheranostics targeting cancer stem cells. Nanomedicine 14 (18), 2487–2514. 10.2217/nnm-2018-0443 31490100

[B58] Regev A. Teichmann S. A. Lander E. S. Amit I. Benoist C. Birney E. (2017). The Human Cell Atlas. eLife 6, e27041. 10.7554/eLife.27041 29206104 PMC5762154

[B59] Rodallec A. Fanciullino R. Lacarelle B. Ciccolini J. (2018). Seek and destroy: improving PK/PD profiles of anticancer agents with nanoparticles. Expert Rev. Clin. Pharmacol. 11 (6), 599–610. 10.1080/17512433.2018.1477586 29768060

[B60] Sørlie T. Perou C. M. Tibshirani R. Aas T. Geisler S. Johnsen H. (2001). Gene expression patterns of breast carcinomas distinguish tumor subclasses with clinical implications. Proc. Natl. Acad. Sci. 98 (19), 10869. 10.1073/pnas.191367098 11553815 PMC58566

[B61] Sato R. Semba T. Saya H. Arima Y. (2016). Concise Review: Stem Cells and Epithelial-Mesenchymal Transition in Cancer: Biological Implications and Therapeutic Targets. Stem Cells 34 (8), 1997–2007. 10.1002/stem.2406 27251010

[B62] Savage P. (2016). Chemotherapy curable malignancies and cancer stem cells: a biological review and hypothesis. BMC Cancer 16 (1), 906. 10.1186/s12885-016-2956-z 27871274 PMC5117562

[B63] Saydam O. Shen Y. Wurdinger T. Senol O. Boke E. James M. F. (2009). Downregulated microRNA-200a in meningiomas promotes tumor growth by reducing E-cadherin and activating the Wnt/beta-catenin signaling pathway. Mol. Cell Biol. 29 (21), 5923–5940. 10.1128/MCB.00332-09 19703993 PMC2772747

[B64] Saygin C. Matei D. Majeti R. Reizes O. Lathia J. D. (2019). Targeting Cancer Stemness in the Clinic: From Hype to Hope. Cell Stem Cell 24 (1), 25–40. 10.1016/j.stem.2018.11.017 30595497

[B65] Schlingensiepen K. H. Jaschinski F. Lang S. A. Moser C. Geissler E. K. Schlitt H. J. (2011). Transforming growth factor-beta 2 gene silencing with trabedersen (AP 12009) in pancreatic cancer. Cancer Sci. 102 (6), 1193–1200. 10.1111/j.1349-7006.2011.01917.x 21366804

[B66] Shibue T. Weinberg R. A. (2017). EMT, CSCs, and drug resistance: the mechanistic link and clinical implications. Nat. Rev. Clin. Oncol. 14 (10), 611–629. 10.1038/nrclinonc.2017.44 28397828 PMC5720366

[B67] Shyu A. B. Wilkinson M. F. van Hoof A. (2008). Messenger RNA regulation: to translate or to degrade. EMBO J. 27 (3), 471–481. 10.1038/sj.emboj.7601977 18256698 PMC2241649

[B68] Song J. Shi W. (2018). The concomitant apoptosis and EMT underlie the fundamental functions of TGF-beta. Acta Biochim. Biophys. Sin. (Shanghai) 50 (1), 91–97. 10.1093/abbs/gmx117 29069287

[B69] Steinbichler T. B. Dudás J. Skvortsov S. Ganswindt U. Riechelmann H. Skvortsova I.-I. (2018). Therapy resistance mediated by cancer stem cells. Semin. Cancer Biol. 53, 156–167. 10.1016/j.semcancer.2018.11.006 30471331

[B70] Sun M. Song H. Wang S. Zhang C. Zheng L. Chen F. (2017). Integrated analysis identifies microRNA-195 as a suppressor of Hippo-YAP pathway in colorectal cancer. J. Hematol. Oncol. 10 (1), 79. 10.1186/s13045-017-0445-8 28356122 PMC5372308

[B71] Tanabe S. Ono R. (2018). The gene and microRNA networks of stem cells and reprogramming. AIMS Cell Tissue Eng. 2, 238–245. 10.3934/celltissue.2018.4.238

[B72] Tanabe S. Aoyagi K. Yokozaki H. Sasaki H. (2014). Gene expression signatures for identifying diffuse-type gastric cancer associated with epithelial-mesenchymal transition. Int. J. Oncol. 44 (6), 1955–1970. 10.3892/ijo.2014.2387 24728500

[B73] Tanabe S. Aoyagi K. Yokozaki H. Sasaki H. (2015a). Regulated genes in mesenchymal stem cells and gastric cancer. World J. Stem Cells 7 (1), 208–222. 10.4252/wjsc.v7.i1.208 25621121 PMC4300932

[B74] Tanabe S. Komatsu M. Kazuhiko A. Yokozaki H. Sasaki H. (2015b). Implications of epithelial-mesenchymal transition in gastric cancer. Trans. Gastrointestinal Cancer 4 (4), 258–264. 10.3978/j.issn.2224-4778.2015.07.04

[B75] Tanabe S. Aoyagi K. Yokozaki H. Sasaki H. (2018). Molecular pathway network of EFNA1 in cancer and mesenchymal stem cells. AIMS Cell Tissue Eng. 2, 58–77. 10.3934/celltissue.2018.2.58

[B76] Tanabe S. (2015a). Origin of cells and network information. World J. Stem Cells 7 (3), 535–540. 10.4252/wjsc.v7.i3.535 25914760 PMC4404388

[B77] Tanabe S. (2015b). Signaling involved in stem cell reprogramming and differentiation. World J. Stem Cells 7 (7), 992–998. 10.4252/wjsc.v7.i7.992 26328015 PMC4550631

[B78] Tanabe S. (2017). Molecular markers and networks for cancer and stem cells. J. Embryol. Stem Cell Res. 1 (1), 000101.

[B79] Tanabe S. (2018a). Molecular Network and Cancer. Res. J. Oncol. 2 (1), 3.

[B80] Tanabe S. (2018b). Wnt Signaling and Epithelial-Mesenchymal Transition Network in Cancer. Res. J. Oncol. 2 (2), 3.

[B81] Tanaka H. Shinto O. Yashiro M. Yamazoe S. Iwauchi T. Muguruma K. (2010). Transforming growth factor beta signaling inhibitor, SB-431542, induces maturation of dendritic cells and enhances anti-tumor activity. Oncol. Rep. 24 (6), 1637–1643. 10.3892/or_00001028 21042762

[B82] Terry S. Chouaib S. (2015). EMT in immuno-resistance. Oncoscience 2 (10), 841–842. 10.18632/oncoscience.226 26682272 PMC4671947

[B83] Tran S. DeGiovanni P. J. Piel B. Rai P. (2017). Cancer nanomedicine: a review of recent success in drug delivery. Clin. Transl. Med. 6 (1), 44. 10.1186/s40169-017-0175-0 29230567 PMC5725398

[B84] Tsubakihara Y. Moustakas A. (2018). Epithelial-Mesenchymal Transition and Metastasis under the Control of Transforming Growth Factor β. Int. J. Mol. Sci. 19 (11), 3672. 10.3390/ijms19113672 PMC6274739 30463358

[B85] van Gils N. Verhagen H. Smit L. (2017). Reprogramming acute myeloid leukemia into sensitivity for retinoic-acid-driven differentiation. Exp. Hematol. 52, 12–23. 10.1016/j.exphem.2017.04.007 28456748

[B86] Visvader J. E. Lindeman G. J. (2008). Cancer stem cells in solid tumours: accumulating evidence and unresolved questions. Nat. Rev. Cancer 8 (10), 755–768. 10.1038/nrc2499 18784658

[B87] Wahl G. M. Spike B. T. (2017). Cell state plasticity, stem cells, EMT, and the generation of intra-tumoral heterogeneity. NPJ Breast Cancer 3, 14. 10.1038/s41523-017-0012-z 28649654 PMC5460241

[B88] Wang M. Miura Y. Tsuchihashi K. Miyano K. Nagano O. Yoshikawa M. (2016). Eradication of CD44-variant positive population in head and neck tumors through controlled intracellular navigation of cisplatin-loaded nanomedicines. J. Controlled Release 230, 26–33. 10.1016/j.jconrel.2016.03.038 27040816

[B89] Weidenfeld K. Schif-Zuck S. Abu-Tayeh H. Kang K. Kessler O. Weissmann M. (2016). Dormant tumor cells expressing LOXL2 acquire a stem-like phenotype mediating their transition to proliferative growth. Oncotarget 7 (44), 71362–71377. 10.18632/oncotarget.12109 27655685 PMC5342084

[B90] Wendt M. K. Smith J. A. Schiemann W. P. (2010). Transforming growth factor-beta-induced epithelial-mesenchymal transition facilitates epidermal growth factor-dependent breast cancer progression. Oncogene 29 (49), 6485–6498. 10.1038/onc.2010.377 20802523 PMC3076082

[B91] Yan Y. Zuo X. Wei D. (2015). Concise Review: Emerging Role of CD44 in Cancer Stem Cells: A Promising Biomarker and Therapeutic Target. Stem Cells Transl. Med. 4 (9), 1033–1043. 10.5966/sctm.2015-0048 26136504 PMC4542874

[B92] Yang M.-H. Wu M.-Z. Chiou S.-H. Chen P.-M. Chang S.-Y. Liu C.-J. (2008). Direct regulation of TWIST by HIF-1α promotes metastasis. Nat. Cell Biol. 10, 295. 10.1038/ncb1691 18297062

[B93] Zhang J. Ma L. (2012). MicroRNA control of epithelial-mesenchymal transition and metastasis. Cancer Metastas. Rev. 31 (3-4), 653–662. 10.1007/s10555-012-9368-6 PMC3686549 22684369

[B94] Zhang J. Tian X. J. Xing J. (2016). Signal Transduction Pathways of EMT Induced by TGF-beta, SHH, and WNT and Their Crosstalks. J. Clin. Med. 5 (4), 41. 10.3390/jcm5040041 PMC4850464 27043642

